# 5α-reductase 1 mRNA levels are positively correlated with TRAMP mouse prostate most severe lesion scores

**DOI:** 10.1371/journal.pone.0175874

**Published:** 2017-05-11

**Authors:** Alexander B. Opoku-Acheampong, Jamie N. Henningson, Amanda P. Beck, Brian L. Lindshield

**Affiliations:** 1 Department of Food, Nutrition, Dietetics and Health, Kansas State University, Manhattan, KS, United States of America; 2 College of Veterinary Medicine, Kansas State University, Manhattan, KS, United States of America; 3 Department of Pathology, Albert Einstein College of Medicine, Bronx, NY, United States of America; University of Minnesota Hormel Institute, UNITED STATES

## Abstract

**Background:**

The contribution of 5α-reductase 1 and 5α-reductase 2 to prostate cancer development and progression is not clearly understood. TRAMP mice are a common prostate cancer model, in which 5α-reductase 1 and 5α-reductase 2 expression levels, along with prostate lesions scores, have not been investigated at different time points to further understand prostate carcinogenesis.

**Method/Principal findings:**

To this end, 8-, 12-, 16-, and 20-week-old male C57BL/6TRAMP x FVB mice prostate most severe and most common lesion scores, 5α-reductase 1 and 5α-reductase 2 in situ hybridization expression, and Ki-67, androgen receptor, and apoptosis immunohistochemistry levels were measured. Levels of these markers were quantified in prostate epithelium, hyperplasia, and tumors sections. Mice developed low- to high-grade prostatic intraepithelial neoplasia at 8 weeks as the most severe and most common lesions, and moderate- and high-grade prostatic intraepithelial neoplasia at 12 and 16 weeks as the most severe lesion in all lobes. Moderately differentiated adenocarcinoma was observed at 20 weeks in all lobes. Poorly differentiated carcinoma was not observed in any lobe until 12-weeks-old. 5α-reductase 1 and 5α-reductase 2 were not significantly decreased in tumors compared to prostate epithelium and hyperplasia in all groups, while proliferation, apoptosis, and androgen receptor were either notably or significantly decreased in tumors compared with prostate epithelium and hyperplasia in most or all groups. Prostate 5αR1 levels were positively correlated with adjusted prostate most severe lesion scores.

**Conclusion:**

Downregulation of androgen receptor and 5α-reductase 2, along with upregulation of 5α-reductase 1 in tumors may promote prostatic intraepithelial neoplasia and prostate cancer development in TRAMP mice.

## Introduction

Among US men, prostate cancer is the most commonly diagnosed cancer and the third-leading cause of cancer death [1]. Androgens are primarily involved in the development and progression of prostate cancer [2]. Testosterone is converted by the isoenzymes 5α-reductase 1 (5αR1) and 5α-reductase 2 (5αR2) into dihydrotestosterone to regulate the prostate gland [3,4]. Androgens bind to the androgen receptor (AR), with the latter acting as a transcription factor to regulate the expression of androgen response genes that modulate many cellular activities such as proliferation and apoptosis to contribute to prostate cancer development and progression [5]. In the normal prostate, 5α-reductase 2 is the primary isoenzyme [6].

Many studies [7–10] although not all [11–13], have observed increased 5α-reductase 1 and/or decreased 5α-reductase 2 mRNA expression or activity in prostate cancer compared with nonmalignant prostate tissue. Additionally, 5α-reductase 1 and 5α-reductase 2 were observed in 73% and 56%, respectively, of human prostate cancer tissues [12]. Taken together, 5α-reductases may be involved in prostate carcinogenesis [14].

A common model is the transgenic mouse model (TRAMP), which uses the rat prostate-specific promoter probasin to direct the expression of the simian virus 40 (SV40) large and small T antigen to the prostate epithelium to cause cell transformation. TRAMP mice develop progressive forms of prostate cancer with lesions ranging from mild prostatic intraepithelial neoplasia (PIN) through well-differentiated (WD) adenocarcinoma, to invasive PD adenocarcinoma with distant site metastasis to pelvic lymph nodes and lungs [15,16]. In TRAMP mice, the transgene is detected as early as 3 weeks of age [17], with pathological features similar to low-grade PIN developing as early as 4–6 weeks of age [18]. C57BL/6 TRAMP x FVB mice develop epithelial hyperplasia by 8 weeks [19], PIN and WD adenocarcinoma by 12 weeks, and invasive carcinoma appearing between 18–30 weeks and metastasizing into lymph nodes and lungs and occasionally kidney, bone and adrenal glands [18,20]. By 30 weeks of age, TRAMP mice on an FVB background (TRAMP x FVB) display 100% metastasis to lungs and lymph nodes along with bone metastasis [16,21].

In addition to not understanding 5α-reductase levels in TRAMP mice, modified grading schemes [22] have not been employed to assess the development and progression of prostate cancer in these mice. Thus, this study was designed to quantify the cell-type specific expression patterns of 5αR1 and 5αR2 mRNA, AR and Ki-67 protein, along with apoptotic DNA fragmentation, in prostate tissue sections of 8-, 12-, 16-, and 20-week-old C57BL/6 TRAMP x FVB mice to characterize how their levels vary at different ages and stages of prostate carcinogenesis. Sixteen weeks is intermediate between 12 and 20 weeks when TRAMP mice would have developed PD and invasive carcinoma [20]. These time points were used previously to study the incidence and distribution of pathologic changes within each lobe of C57BL/6 TRAMP x FVB mice prostate as a function of time [20]. To the best of our knowledge, this study is the first to grade lesions and quantify 5α-reductase levels at different ages in TRAMP mouse prostate.

## Materials and methods

### Ethics statement

The Institutional Animal Care and Use Committee (IACUC) at Kansas State University approved all animal procedures (protocol 2969).

### Study mice and design

Mice were bred as described previously [23]. Male C57BL/6 TRAMP x FVB mice were weaned and began consuming AIN-93G diet at 3 weeks of age before being randomized to 8- (n = 5), 12- (n = 8), 16- (n = 9), and 20- (n = 12) week groups. Mice were cohoused until 8 weeks, and then individually housed, monitored daily, weighed weekly, and provided AIN-93G diet and water *ad libitum*. Mice were terminated at their respective study time points by anesthetizing via CO_2_ inhalation and euthanized by exsanguination. The genitourinary (GU) tracts, kidneys, and lungs were dissected, and the GU tracts were weighed. Iliac lymph nodes were also collected whenever possible. All tissues were fixed by immersion in 10% neutral buffered formalin for 48 hours, and then moved to 70% alcohol until processing in the Kansas State University Veterinary Diagnostic Laboratory.

### Histopathology

Histological processing of prostate tissues, sectioning, scoring for most common and most severe lesions, and adjusting for distribution, were performed as described previously [22,23]. The anterior, dorsal, lateral and ventral lobes of C57BL/6 TRAMP × FVB mice were assigned two grades each between 0–7. The first grade is the most severe lesion within the lobe [normal prostate as least severe (grade 0), and PD carcinoma as most severe (grade 7)]. The second grade is the most common lesion within the lobe [normal prostate as least common (grade 0), and PD carcinoma as most common (grade 7)]. The most severe and most common lesions within a lobe were adjusted for distribution, either as focal, multifocal, or diffuse. With focal, there are fewer than three foci within the lobe. Multifocal indicates there are three or more foci within the lobe, with less than 50% of the lobe containing the lesion of interest. Diffuse indicates greater than 50% of the lobe is affected [23]. Prostate lesions were scored blindly twice by a board-certified pathologist (JNH). Select tissues were also scored blindly twice by a board-certified second veterinary pathologist (APB) to ensure scoring consistency. Iliac lymph nodes examination for metastases was done as described previously [23]. Formalin-fixed, paraffin-embedded prostate tissue sections (4 μm) of 8-, 12-, 16-, and 20-week-old (n = 5) AIN-93G-fed male C57BL/6 TRAMP x FVB mice were prepared for biomarker detection. Most prostate samples at the time of euthanasia were completely obliterated by tumor and therefore in most samples, prostate lobes could not be identified. In some instances, all four lobes were replaced by tumor.

### 5α-reductase 1 and 5α-reductase 2 in situ hybridization

5αR1 and 5αR2 accessions NM 175283.3 and NM 053188.2, respectively, obtained from National Center for Biotechnology Information (NCBI) website were submitted to Advanced Cell Diagnostics, Inc., [(ACD), Hayward, CA)] for custom probe design and synthesis for use in their RNAscope assay. Prostate sections were baked in a Fisher Scientific (model 77) slide warmer preheated to 55°C for 25 minutes. Sections were deparaffinized in xylene, rehydrated in 100% ethanol, and air-dried for 5 minutes at room temperature. Sections were then treated serially with: Pre-Treatment 1 solution (endogenous hydrogen peroxidase block with Pretreat 1 solution for 10 minutes at room temperature); Pre-Treatment 2 (100–104°C, 25 minutes immersion in Pretreat 2 solution); and, Pre-Treatment 3 (protease digestion, 40°C for 28 minutes); rinses with distilled water (2X) were performed after each Pre-Treatment step. Sections were then hybridized in 5αR1 or 5αR2 probes, without a cover slip, at 40°C for 2 hours in a HybEZ Oven (ACD). After wash buffer steps, signal amplification from the hybridized probes were performed by the serial application of Amp 1, 40°C for 30 minutes (PreAmplifier step); Amp 2, 40°C for 15 minutes (signal enhancer step); Amp 3, 40°C for 30 minutes (amplifier step); Amp 4, 40°C for 15 minutes (Label Probe step); Amp 5, ambient temperature for 30 minutes, and Amp 6, ambient temperature for 15 minutes (signal amplifications steps). Wash buffer steps with Wash Buffer (ACD, proprietary) were performed after each Amp step. Horseradish peroxidase (HRP) activity was then observed by the application of 3,3′-diaminobenzidine (DAB) for 10 minutes at ambient temperature. Sections were then counterstained with Gill’s hematoxylin, dehydrated through graded ethanol [95% (1X) and 100% (2X)] and xylene and then mounted. Sections were quality controlled for RNA integrity with an RNAscope probe for cyclophilin B (*Mm*-*Ppib*) RNA (positive control) and for nonspecific background with a probe for bacterial *dap*B RNA (negative control). Specific RNA staining signal was identified as brown, punctate dots.

### Immunohistochemistry

#### Androgen receptor

Prostate sections were baked in a Fisher Scientific Isotemp (model 281A) vacuum oven preheated to 60°C for 30 minutes. After baking, sections were deparaffinized in xylene and rehydrated using a decreasing ethanol gradient [100% (2X), 95% and 80% (1X)]. Endogenous alkaline phosphatase was blocked using 3% hydrogen peroxide in methanol. Antigen retrieval was carried out by microwaving at 540W in 10 mM Tris/1 mM EDTA buffer, pH 9, four times for 5 minutes. Sections were blocked with 2.5% normal horse serum (Vector Laboratories, Inc., Burlingame, CA) before incubation for 1 hour at 37°C with a rabbit polyclonal antibody to AR (1:50 dilution; N-20, sc-816; Santa Cruz Biotechnology, Santa Cruz, CA). After washing, sections were incubated with ImmPRESS-AP anti-rabbit IgG (alkaline phosphatase) polymer detection reagent (MP-5401; Vector Laboratories, Inc.,) for 30 minutes at room temperature. Colors were developed with a Vector Red alkaline phosphatase substrate kit (SK-5100; Vector Laboratories, Inc.,). Slides were subsequently counterstained with hematoxylin (Vector Laboratories, Inc.,), and processed back to xylene through an increasing ethanol gradient [80% and 95% (1X), and 100% (2X)] and then mounted. Normal rabbit IgG (1:100; sc-2027: Santa Cruz Biotechnology) was used as a negative control.

#### Ki-67

Prostate sections were deparaffinized in Leica Bond Dewax Solution (Leica Microsystems Inc., Buffalo Grove, IL) at 72°C, and then rehydrated in 100% ethanol, followed by 3 washes in Tris-buffered saline. Antigen retrieval was carried out with Novocastra Bond Epitope Retrieval Solution 1 (citrate buffer, pH 6) (AR9961; Leica Microsystems Inc.,) for 20 minutes at 100°C. The sections were stained with a prediluted rabbit monoclonal antibody to Ki-67 (CPRM325AA; Biocare Medicals, Concord, CA) for 15 minutes at room temperature. The anti-rabbit poly-HRP-IgG polymer from the Bond Polymer Refine Detection System (Leica Microsystems Inc.,) was used for the enhancement of the signal. The substrate chromogen, DAB, was used for the detection of the complex. Sections were counterstained with Gill’s hematoxylin, and processed back to xylene through an increasing ethanol gradient [95% (1X) and 100% (2X)], and then mounted. Tissue from a canine mast cell tumor was used as a positive control.

#### Apoptosis

Terminal deoxynucleotidyl transferase dUTP nick end labeling (TUNEL) staining was performed using the Apoptag Peroxidase *In Situ* Apoptosis Detection kit (S7100; Millipore, Temecula, CA). Prostate sections were baked in a Fisher Scientific Isotemp (model 281A) vacuum oven preheated to 60°C for 30 minutes. After baking, sections were deparaffinized in xylene, rehydrated through a graded ethanol series [100% (2X), 95% and 70% (1X)] and treated with ready-to-use proteinase K (S302080-2; Dako North America, Inc., Carpinteria, CA) for 15 minutes at room temperature. Sections were washed with 2 changes of distilled water for 5 minutes each. Endogenous peroxidases were blocked with 3% hydrogen peroxide in phosphate buffered saline (PBS, pH 7.4), for 5 minutes and washed with 2 changes of PBS. Equilibration buffer containing digoxigenin-conjugated nucleotides was placed directly onto the section for 10 seconds. Sections were incubated with terminal deoxynucleotide transferase (TdT) enzyme (1:15 dilution) in a humidified chamber at 37°C for 1 hour. Sections were then incubated for 10 minutes at room temperature in stop-wash buffer, rinsed in 3 changes of PBS for 1 minute each, and then incubated with anti-digoxigenin conjugate for 30 minutes at room temperature. Sections were washed in 4 changes of PBS for 2 minutes each, and then incubated with the substrate chromogen, DAB, for 3 minutes. Sections were then stained with 0.5% (w/v) methyl green counterstain, dehydrated through 100% N-butanol (3X) and xylene and then mounted.

### Biomarker quantification

**Fig 1:**
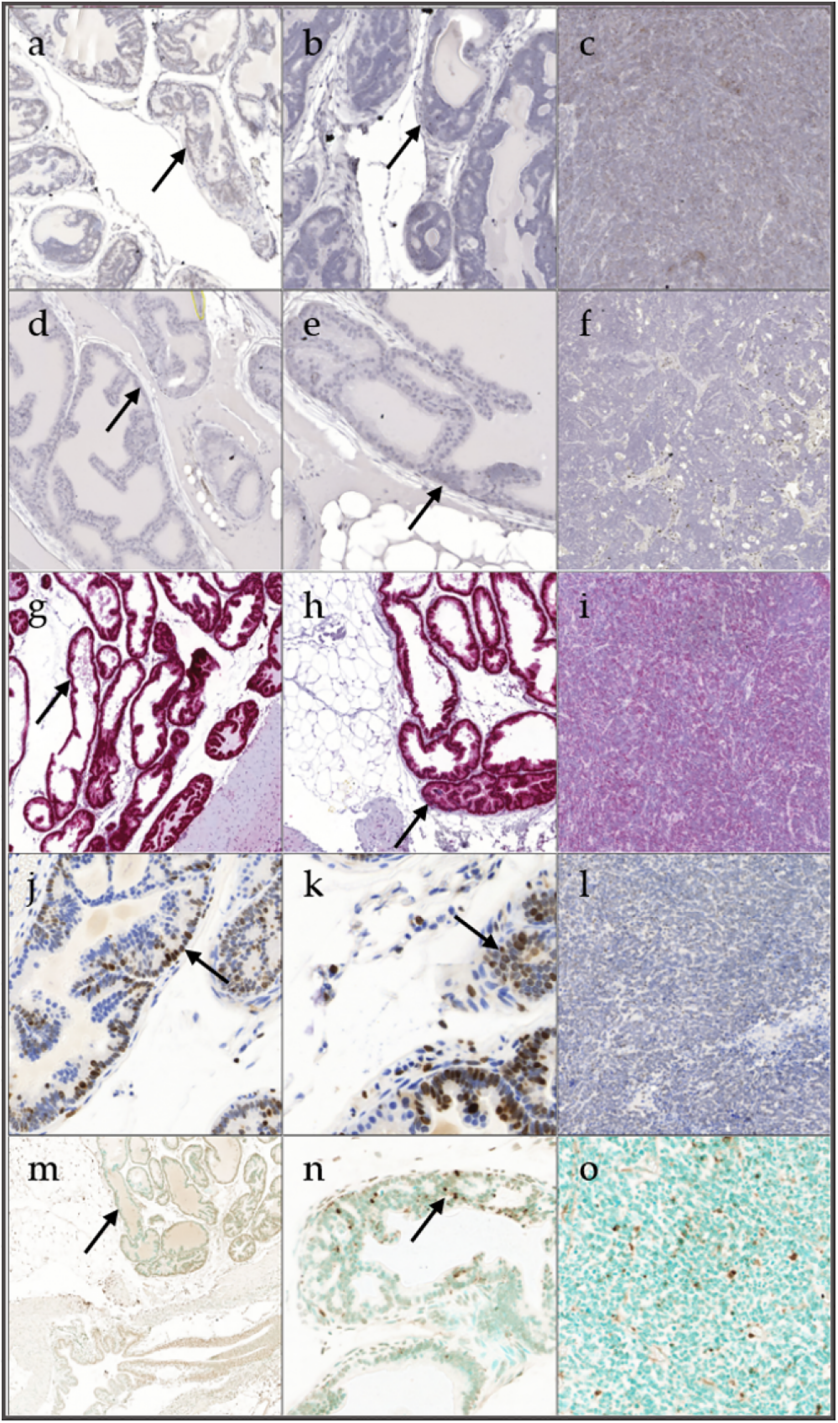
Representative staining for 5α-reductase 1 (a), 5α-reductase 2 (d), androgen receptor (g), Ki-67 (j) and apoptosis (m) in prostate epithelium showing single layer of cells; 5α-reductase 1 (b), 5α-reductase 2 (e), androgen receptor (h), Ki-67 (k) and apoptosis (n) in hyperplasia showing focal increased cell density with piling up on one another; and 5α-reductase 1 (c), 5α-reductase 2 (f), androgen receptor (i), Ki-67 (l) and apoptosis (o) in tumor showing diffuse sheets of cells with no organization and characterized by neoplastic cellular characteristics. Staining in prostate and hyperplasia are shown with a black arrow.

A board-certified pathologist identified areas of epithelium that were composed of a single layer of cells as prostate epithelium; hyperplastic regions as where there was increased density of cells piled up on one another; and tumor as diffuse sheets of cells with no organization characterized by neoplastic cellular characteristics, using an Olympus DP26 digital camera (Olympus America, Center Valley, PA). These areas were scanned using a Pannoramic Midi scanner (3D Histech, Budapest, Hungary) with a 20X objective, giving 2-megapixel resolution. Images were captured using the 3D Histech software. Scanned images were then annotated using Halo software (Indica Laboratory, Coralles, NM). In tumors, areas of viable neoplastic cells away from areas of necrosis were measured. Ki-67 and AR protein, and apoptotic DNA fragmentation were quantified using the double stain cytoplasmic and nuclear immunohistochemistry algorithm (Indica Laboratory), which was set to identify a single positive nuclear stain. 5αR1 and 5αR2 mRNA were quantified using the chromogenic RNA in situ hybridization algorithm (Indica Laboratory), which measures RNA probes on a per cell basis. Data were quantified for Ki-67, AR, apoptosis, 5αR1 and 5αR2 in prostate epithelium, hyperplasia, and tumor of prostate sections. Prostate epithelium was defined as histologically normal prostate. Representative images in Fig 1 were captured at 2X magnification.

The total positive cells per tissue area for Ki-67, AR and apoptosis was calculated as:

Total positive cells per tissue area (μm^2^) = Total positive cells/Tissue area (μm^2^)

The total probe copies per tissue area for 5αR1 and 5αR2 mRNA was calculated as:

Total probe copies per tissue area (μm^2^) = Total probe copies/Total tissue area (μm^2^)

Total pixels per tissue area for 5αR1 and 5αR2 mRNA was also calculated, but this was not used for data analysis due to poor sensitivity. Immunohistochemistry and in situ hybridization data are from a single experiment for each biomarker.

### Statistical analysis

Data were analyzed using ANOVA with Fisher’s Least Significant Difference (LSD), utilizing SAS 9.4 (SAS Institute Inc., Cary, NC) with *p* < 0.05 considered statistically significant. Cell types with n < 2 were excluded from statistical analysis, and the modified Thompson tau technique was used to eliminate outliers. Iliac lymph node metastases incidence was analyzed using the Kruskal Wallis non-parametric one-way ANOVA. Correlation between biomarkers and adjusted prostate severe lesion scores was analyzed using the Spearman rank correlation coefficient (r).

## Results

### GU weights and iliac lymph node metastases

GU weights in 8-, 12-, 16-, and 20-week-old C57BL/6 TRAMP x FVB mice were increased as a function of age (Table 1). Twenty-week-old mice had significantly increased GU weights versus 8-, 12-, and 16-week-old mice. No significant differences were observed in the incidence of iliac lymph node metastases between groups (Table 1); however, a notable difference was found in incidence between 20- (42%) and 16- (11%), 12- (0%) and 8- (0%) week-old mice.

**Table 1:** C57BL/6 TRAMP x FVB mice GU weights^1^ and iliac lymph node metastases incidence.

**Group**	**n**	**GU weights (g)**	**n**	**Lymph node** **metastases incidence (%)**
8-weeks	5	0.30 ± 0.01^a^	2	0 (0)
12-weeks	8	0.38 ± 0.02^a^	8	0 (0)
16-weeks	9	0.62 ± 0.07^a^	9	1 (11)
20-weeks	12	3.88 ± 1.09^b^	12	5 (42)

^1^Data are mean ± SEM; values with different letters are statistically different (*p* < 0.05).

### Most severe and most common lesion scores and histopathological distribution

Anterior and dorsal lobes raw and adjusted mean most severe and most common lesion scores were significantly increased in 20- versus 8-, 12-, and 16-week-old mice (Tables 2 and 3). The dorsal lobe adjusted mean most common lesion score was significantly increased in 16- compared to 12-week-old mice. Lateral and ventral lobes raw and adjusted mean most severe and most common lesion scores were significantly increased in 16- and 20- versus 8- and 12-week-old mice.

**Table 2:** Raw and adjusted mean most severe lesion scores for the anterior, dorsal, lateral, and ventral prostate lobes^1^.

		Anterior prostate		Dorsal prostate		Lateral prostate		Ventral prostate	
Group	n	Raw	Adjusted	Raw	Adjusted	Raw	Adjusted	Raw	Adjusted
8-weeks	5	2.10 ± 0.28^a^	4.10 ± 0.86^a^	2.50 ± 0.17^a^	6.10 ± 0.38^a^	1.90 ± 0.10^a^	4.80 ± 0.47^a^	1.60 ± 0.16^a^	4.30 ± 0.63^a^
12-weeks	8	2.56 ± 0.13^a^	6.13 ± 0.30^a^	3.00 ± 0.29^a^	7.38 ± 0.81^a^	3.00 ± 0.41^a^	7.50 ± 1.15^a^	1.56 ± 0.27^a^	3.56 ± 0.67^a^
16-weeks	9	2.39 ± 0.31^a^	5.78 ± 0.95^a^	3.78 ± 0.42^a^	10.17 ± 1.39^a^	5.17 ± 0.50^b^	14.44 ± 1.62^b^	4.83 ± 0.59^b^	13.66 ± 1.87^b^
20-weeks	12	4.92 ± 0.51^b^	14.21 ± 1.65^b^	5.63 ± 0.43^b^	16.33 ± 1.38^b^	5.42 ± 0.51^b^	15.88 ± 1.62^b^	4.86 ± 0.61^b^	14.18 ± 1.93^b^

^1^Data are mean ± SEM; values with different letters are statistically different from one another (*p* < 0.05).

**Table 3:** Raw and adjusted mean most common lesion scores for the anterior, dorsal, lateral, and ventral prostate lobes^1^.

		Anterior prostate		Dorsal prostate		Lateral prostate		Ventral prostate	
Group	n	Raw	Adjusted	Raw	Adjusted	Raw	Adjusted	Raw	Adjusted
8-weeks	5	1.30 ± 0.21^a^	2.70 ± 0.58^a^	2.0^a^	5.20 ± 0.13^a,b^	1.50 ± 0.17^a^	3.70 ± 0.65^a^	1.60 ± 0.16^a^	4.30 ± 0.63^a^
12-weeks	8	1.81 ± 0.14^a^	4.44 ± 0.41^a^	2.0^a^	5.50 ± 0.26^a^	1.63 ± 0.13^a^	4.25 ± 0.46^a^	1.25 ± 0.21^a^	3.13 ± 0.62^a^
16-weeks	9	1.83 ± 0.34^a^	4.56 ± 1.01^a^	3.22 ± 0.50^a^	9.39 ± 1.49^b^	4.11 ± 0.63^b^	11.94 ± 1.93^b^	4.22 ± 0.60^b^	12.28 ± 1.87^b^
20-weeks	12	4.83 ± 0.53^b^	14.04 ± 1.69^b^	4.75 ± 0.50^b^	14.00 ± 1.55^c^	5.21 ± 0.52^b^	15.25 ± 1.67^b^	4.77 ± 0.63^b^	14.09 ± 1.96^b^

^1^Data are mean ± SEM; values with different letters are statistically different from one another (*p* < 0.05).

Anterior lobe LG-PIN as the most severe lesion was significantly increased in 8- versus 12- and 20-week-old mice (Table 4). LG-PIN as the most severe lesion was significantly increased in the ventral lobe of 8- compared to 16- and 20-week-old mice. Lateral lobe moderate-grade (MG) PIN as the most severe lesion was significantly increased in 8- versus 12-week-old, and in 8- and 12- compared to 16- and 20-week-old mice. MG-PIN as the most severe lesion was significantly increased in the ventral lobe of 8- versus 12-, 16-, and 20-week-old mice. MG-PIN as the most severe lesion was significantly increased in the ventral lobe of 12- and 16- compared to 20-week-old mice. Lateral lobe HG-PIN as the most severe lesion was significantly increased in 12- and 16- versus 8- and 20-week-old mice. HG-PIN as the most severe lesion was significantly increased in the ventral lobe of 12-week-old mice compared to 8- and 20-week-old mice. Dorsal lobe HG-PIN as the most severe lesion was significantly increased in 12- and 16- versus 20-week-old mice. PD carcinoma and prostate cancer as the most severe lesions were significantly increased in the anterior lobe of 20- compared to 8-, 12-, and 16-week-old mice. Dorsal lobe PD carcinoma and prostate cancer as the most severe lesions were significantly increased in 20- versus 8-, 12-, and 16-week-old mice. PD carcinoma and prostate cancer as the most severe lesions were significantly increased in the dorsal lobe of 16- compared to 8- and 12-week-old mice. Lateral and ventral lobes PD carcinoma and prostate cancer as the most severe lesions were significantly increased in 16- and 20- versus 8- and 12-week-old mice. Prostate cancer as the most severe lesion was significantly increased in the lateral and ventral lobes of 20- compared to 16-week-old mice.

**Table 4:** Histopathological analysis (most severe lesion) of individual prostate lobes in 8-, 12-, 16-, and 20-week-old C57BL/6 TRAMP x FVB mice^1^^,^ ^2^.

Prostatic intraepithelial neoplasia					Adenocarcinoma			Prostate cancer
	n	LG	MG	HG	WD	MD	PD	(WD-PD)
**Anterior prostate**								
8-weeks	5	30%^a^	30%	40%	0%	0%	0%^a^	0%^a^
12-weeks	8	0%^b^	44%	56%	0%	0%	0%^a^	0%^a^
16-weeks	9	17%^a,b^	50%	28%	0%	0%	6%^a^	6%^a^
20-weeks	12	8%^b^	8%	17%	0%	4%	54%^b^	58%^b^
**Dorsal prostate**								
8-weeks	5	0%	50%	50%^a,b^	0%	0%	0%^a^	0%^a^
12-weeks	8	0%	25%	69%^a^	0%	0%	6%^a^	6%^a^
16-weeks	9	0%	11%	67%^a^	0%	0%	22%^b^	22%^b^
20-weeks	12	0%	17%	13%^b^	0%	4%	67%^c^	71%^c^
**Lateral prostate**								
8-weeks	5	10%	90%^a^	0%^a^	0%	0%	0%^a^	0%^a^
12-weeks	8	0%	50%^b^	38%^b^	0%	0%	13%^a^	13%^b^
16-weeks	9	0%	6%^c^	39%^b^	0%	0%	56%^b^	56%^c^
20-weeks	12	17%	4%^c^	8%^a^	0%	4%	67%^b^	71%^d^
**Ventral prostate**								
8-weeks	5	40%^a^	60%^a^	0%^a^	0%	0%	0%^a^	0%^a^
12-weeks	8	13%^a,b^	44%^b^	19%^b^	0%	0%	0%^a^	0%^a^
16-weeks	9	0%^b^	39%^b^	6%^a,b^	0%	0%	56%^b^	56%^b^
20-weeks	12	8%^b^	17%^c^	0%^a^	0%	4%	54%^b^	58%^c^

^1^Values with different letters are statistically different from one another (*p* < 0.05).

^2^LG = low-grade, MG = moderate-grade, HG = high-grade, PIN = prostatic intraepithelial neoplasia, WD = well-differentiated, MD = moderately differentiated, PD = poorly differentiated.

Anterior lobe LG-PIN as the most common lesion was significantly increased in 8- versus 12- and 20-week-old mice (Table 5). MG-PIN as the most common lesion was significantly increased in the anterior lobe of 12- compared to 8-, 16-, and 20-week-old mice. Dorsal lobe MG-PIN as the most common lesion was significantly increased in 8- and 12- versus 16- and 20-week-old mice. Ventral lobe MG-PIN as the most common lesion was significantly increased in 8-, 12-, and 16- compared to 20-week-old mice. Dorsal lobe HG-PIN as the most common lesion was significantly increased in 16- and 20- versus 8- and 12-week-old mice. HG-PIN as the most common lesion was significantly increased in the dorsal lobe of 16- compared to 20-week-old mice. Anterior and dorsal lobes PD carcinoma and prostate cancer as the most common lesions were significantly increased in 20- versus 8-, 12- and 16-week-old mice. PD carcinoma as the most common lesion was significantly increased in the dorsal lobe of 20- compared to 16-week-old mice. PD carcinoma and prostate cancer as the most common lesions were significantly increased in the lateral and ventral lobes of 20- versus 16-week-old mice. Lateral and ventral lobes PD carcinoma and prostate cancer as the most common lesions were significantly increased in 16- and 20- compared to 8- and 12-week-old mice.

**Table 5:** Histopathological analysis (most common lesion) of individual prostate lobes in 8-, 12-, 16-, and 20-week-old C57BL/6 TRAMP x FVB mice^1^^,^ 2.

Prostatic intraepithelial neoplasia					Adenocarcinoma			Prostate cancer
	n	LG	MG	HG	WD	MD	PD	(WD-PD)
**Anterior prostate**								
8-weeks	5	80%^a^	10%^a^	10%	0%	0%	0%^a^	0%^a^
12-weeks	8	25%^b,c^	69%^b^	6%	0%	0%	0%^a^	0%^a^
16-weeks	9	50%^a,b^	39%^a^	6%	0%	0%	6%^a^	6%^a^
20-weeks	12	13%^c^	21%^a^	8%	0%	4%	54%^b^	58%^b^
**Dorsal prostate**								
8-weeks	5	0%	100%^a^	0%^a^	0%	0%	0%^a^	0%^a^
12-weeks	8	0%	100%^a^	0%^a^	0%	0%	0%^a^	0%^a^
16-weeks	9	0%	67%^b^	11%^b^	0%	0%	22%^b^	22%^a^
20-weeks	12	0%	38%^b^	8%^c^	0%	4%	50%^c^	54%^b^
**Lateral prostate**								
8-weeks	5	50%	50%	0%	0%	0%	0%^a^	0%^a^
12-weeks	8	38%	63%	0%	0%	0%	0%^a^	0%^a^
16-weeks	9	11%	44%	0%	0%	0%	44%^b^	44%^b^
20-weeks	12	17%	8%	8%	0%	4%	63%^c^	67%^c^
**Ventral prostate**								
8-weeks	5	40%	60%^a^	0%	0%	0%	0%^a^	0%^a^
12-weeks	8	25%	50%^a^	0%	0%	0%	0%^a^	0%^a^
16-weeks	9	0%	56%^a^	0%	0%	0%	44%^b^	44%^b^
20-weeks	12	17%	8%^b^	0%	0%	4%	54%^c^	58%^c^

^1^Values with different letters are statistically different from one another (*p* < 0.05).

^2^LG = low-grade, MG = moderate-grade, HG = high-grade, PIN = prostatic intraepithelial neoplasia, WD = well-differentiated, MD = moderately differentiated, PD = poorly differentiated.

### 5αR1, 5αR2, AR, proliferation, and apoptosis

Prostate epithelium, hyperplasia and tumor representative stainings for 5αR1 are shown in Fig 1A, 1B and 1C, respectively. Prostate epithelium, hyperplasia and tumor representative stainings for 5αR2 are shown in Fig 1D, 1E and 1F, respectively. There was no significant difference in 5αR1 and 5αR2 in the cell types within and between groups (Tables 6 and 7). 5αR1 was predominantly expressed in prostate epithelium versus stroma while 5αR2 was highly expressed in both prostate epithelium and stroma. Prostate epithelium, hyperplasia and tumor AR representative stainings are shown in Fig 1G, 1H and 1I, respectively. There was a significant increase in AR expression in hyperplasia compared to prostate epithelium and tumor of 16-week-old mice (Table 8). There was a significant decrease in AR expression in tumor versus prostate epithelium and hyperplasia of 20-week-old mice. Prostate epithelium, hyperplasia and tumor Ki-67 representative stainings are shown in Fig 1J, 1K and 1L, respectively. There was a significant increase in Ki-67 expression in prostate epithelium and hyperplasia of 8- compared to 16- and 20-week-old mice (Table 9). There was a significant increase in Ki-67 expression in prostate epithelium of 12- versus 16-week-old mice. There was a significant increase in Ki-67 expression in hyperplasia compared to prostate epithelium and tumor of 20-week-old mice. Prostate epithelium, hyperplasia and tumor apoptosis representative stainings are shown in Fig 1M, 1N and 1O, respectively. There was no significant difference in apoptosis in the cell types within and between groups (Table 10).

**Table 6:** 5α-reductase 1 expression in 8-, 12-, 16-, and 20-week-old C57BL/6 TRAMP x FVB mice. The values are the mean total 5α-reductase 1 probe copies per tissue area (μm^2^) ± SEM in prostate epithelium, hyperplasia or tumor.

**5α-reductase 1**						
**Group**	n	Prostate epithelium	n	Hyperplasia	n	Tumor
8-weeks	5	5.8 ± 2.5	5	5.4 ± 1.1	-	—
12-weeks	4	18.7 ± 8.7	5	10.5 ± 4.1	2	16.8 ± 12.1
16-weeks	5	15.1 ± 5.3	5	6.7 ± 3.5	2	15.7 ± 5.1
20-weeks	2	22.4 ± 9.5	2	13.5 ± 5.6	4	15.8 ± 4.1

*p* < 0.05 *vs*. prostate epithelium, hyperplasia and tumor within and between groups. Data are multiplied by 1000.

**Table 7:** 5α-reductase 2 expression in 8-, 12-, 16-, and 20-week-old C57BL/6 TRAMP x FVB mice. The values are the mean total 5α-reductase 2 probe copies per tissue area (μm^2^) ± SEM in prostate epithelium, hyperplasia or tumor.

**5α-reductase 2**						
**Group**	n	Prostate epithelium	n	Hyperplasia	n	Tumor
8-weeks	5	9.0 ± 4.7	5	3.8 ± 1.3	-	—
12-weeks	5	12.2 ± 5.3	5	9.0 ± 4.6	2	12.7 ± 10.6
16-weeks	4	7.5 ± 2.6	4	6.9 ± 3.6	2	0.7
20-weeks	1	17.7	1	1.8	3	4.1 ± 1.6

*p* < 0.05 *vs*. prostate epithelium, hyperplasia and tumor within and between groups. Data are multiplied by 1000.

**Table 8:** Androgen receptor (AR) expression in 8-, 12-, 16-, and 20-week-old C57BL/6 TRAMP x FVB mice. The values are the mean total AR positive cells per tissue area (μm^2^) ± SEM in prostate epithelium, hyperplasia or tumor.

**Androgen receptor**						
**Group**	n	Prostate epithelium	n	Hyperplasia	n	Tumor
8-weeks	5	5.9 ± 1.1	5	7.1 ± 0.7	-	—
12-weeks	5	7.9 ± 0.6	5	8.5 ± 0.2	1	0.1
16-weeks	5	6.7 ± 0.5^1^	5	9.3 ± 0.9^2^	2	4.0 ± 0.2^1^
20-weeks	5	7.5 ± 1.2^1^	5	9.2 ± 1.3^1^	5	1.5 ± 0.8^2^

*p* < 0.05 *vs*. prostate epithelium, hyperplasia and tumor within group (number). Data are multiplied by 1000.

**Table 9:** Ki-67 expression in 8-, 12-, 16-, and 20-week-old C57BL/6 TRAMP x FVB mice. The values are the mean total Ki-67 positive cells per tissue area (μm^2^) ± SEM in prostate epithelium, hyperplasia or tumor.

**Ki-67**						
**Group**	n	Prostate epithelium	n	Hyperplasia	n	Tumor
8-weeks	5	1.6 ± 0.5^a^	5	3.3 ± 0.9^a^	-	—
12-weeks	5	1.2 ± 0.4^a,b^	5	1.6 ± 0.6^a,b^	1	0.5
16-weeks	5	0.2 ± 0.2^c^	5	0.4 ± 0.3^b^	2	0.2 ± 0.1
20-weeks	5	0.3 ± 0.2^b,c,1^	5	1.2 ± 0.2^b,2^	5	0.3 ± 0.1^1^

*p* < 0.05 *vs*. prostate epithelium, hyperplasia and tumor within (number) and between groups (letter). Data are multiplied by 1000.

**Table 10:** Apoptosis expression in 8-, 12-, 16-, and 20-week-old C57BL/6 TRAMP x FVB mice. The values are the mean total apoptosis positive cells per tissue area (μm^2^) ± SEM in prostate epithelium, hyperplasia or tumor.

**Apoptosis**						
**Group**	n	Prostate epithelium	n	Hyperplasia	n	Tumor
8-weeks	-	—	5	0.4 ± 0.1	-	—
12-weeks	-	—	5	0.8 ± 0.5	1	0.1
16-weeks	-	—	5	0.5 ± 0.2	2	0.2
20-weeks	-	—	4	0.1 ± 0.1	5	0.2 ± 0.1

*p* < 0.05 *vs*. prostate epithelium, hyperplasia and tumor within and between groups. Data are multiplied by 1000.

### Biomarker correlation with adjusted prostate severe lesion scores

**Fig 2:**
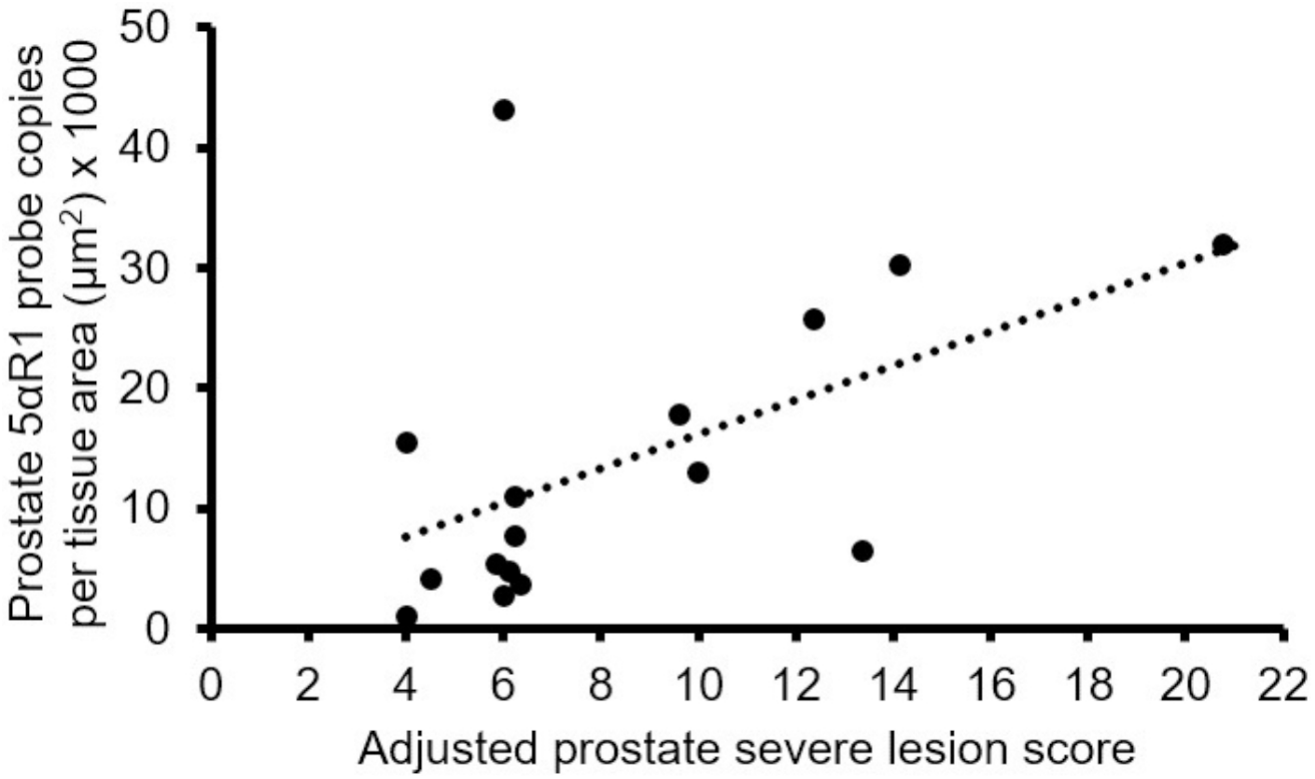
Spearman correlation coefficient (r) between 5α-reductase 1 (5αR1) in prostate and adjusted prostate most severe lesion scores. There was a significant positive correlation between 5α-reductase 1 in prostate and adjusted prostate most severe lesion scores (r = 0.52; *p* = 0.04).

Only one significant correlation between biomarkers and adjusted prostate severe lesion scores was identified. Prostate 5αR1 levels were positively correlated with adjusted prostate most severe lesion scores (Fig 2). Correlation between 5αR1 in prostate and adjusted prostate severe lesion scores was compared in 8-, 12-, 16-, and 20-week-old mice and found to have a similar trend in all groups, thus age did not appear to impact this correlation. Adjusted prostate severe lesion scores was used because it combines lesion severity with an indication of its distribution within a lobe [22].

## Discussion

The raw and adjusted, mean most severe lesion scores were slightly increased in 20-week-old mice compared to 20-week-old control C57BL/6 TRAMP x FVB mice in our previous study [23], which may suggest that in this study, lesions were more severe or progressing faster. Given that this study was conducted at the same time using littermates of that study, this outcome was surprising. Overall GU weights, development and progression of prostate cancer were similar to that reported previously [20,22,24], but contrary to another reported previously [19]. We observed a notable increase in iliac lymph node metastases incidence with increasing age.

5αR1 was predominantly expressed in prostate epithelium, while 5αR2 was highly expressed in both prostate epithelium and stroma consistent to expression in human prostate [11,25]. 5αR1 levels in tumors were not statistically different from prostate epithelium and hyperplasia, which is different than the significantly higher 5αR1 in human prostate cancer compared to normal and/or hyperplastic prostate [11,26]. Although 5αR1 was increased in high grade compared to low grade prostate cancer [27,28], this is the first study to report that prostate 5αR1 levels are positively correlated with prostate most severe lesion scores. 5αR2 in tumors was not statistically different between prostate epithelium and hyperplasia, which is different than the higher 5αR2 levels in benign prostatic hyperplasia (BPH) compared to prostate cancer [9]. Part of this difference might be the difference between BPH and prostate epithelium and hyperplasia.

The marked decrease in AR expression in tumors compared to hyperplasia at 16 weeks, and compared to prostate epithelium and hyperplasia at 20 weeks are consistent with weak to negative AR levels in C57BL/6 TRAMP x FVB mice PD macroscopic tumors regardless of age [29], weak AR staining in C57BL/6 TRAMP x FVB mice MD and PD tumors [20], or absence of AR in most human PD tumors [30]. The loss of AR expression in tumors may be due to them bypassing AR pathway for growth, or a reduction in the stability of AR protein that decreases the AR protein levels to one difficult to detect immunohistologically [31].

The increased Ki-67 levels in hyperplasia, and the overall lack of difference between hyperplasia and tumor Ki-67 levels for all except the 20-week group may suggest that proliferation does not vary much in the model at different time points. The lack of significant difference in tumor cell proliferation between groups is consistent with results seen in the dorsal and ventral prostate lobes of 10-, 16-, and 24-week-old TRAMP mice [32], and tumor between low-lycopene diets at 12, 16, and 20 weeks of age in C57BL/6 TRAMP x FVB mice [24], but not consistent with the significant decrease in tumor of 17-, 24-, and 36-week-old C57BL/6 TRAMP mice with age [33]. All mice expressed Ki-67 in predominantly PD tumors, which is greater than ~70% of C57BL/6 TRAMP x FVB mice that expressed Ki-67 in PD tumors in a previous study [20].

Apoptosis was completely absent in the prostate epithelium, probably due to the strong oncogenic pressure present in these mice with an SV40 Tag–induced disturbance in p53- and Rb-regulated cell functions [18,34]. Higher apoptosis in hyperplasia compared to tumor is consistent with elevated apoptosis in preneoplastic lesions [35]. Though not statistically significant, increased tumor apoptosis with age is consistent with a similar finding in the dorsolateral prostate of 17-, 24-, and 36-week-old C57BL/6 TRAMP mice [33]. Apoptosis was lower in prostate epithelium than tumor which is not consistent with the progressive decrease in apoptosis from BPH, high-grade PIN, to prostate cancer [36]. Conversely, there were no significant differences in apoptosis in all lobes between low-lycopene diets at 12, 16, and 20 weeks of age in C57BL/6 TRAMP x FVB mice [24].

There are limitations to this study that should be noted. We were unable to make some comparisons due to the lack of replicates or loss of one or more cell types. This limited the interpretation of the results. In addition, the fact that many of these tumors are of neuroendocrine origin suggests that results may be applicable to a subpopulation of patients with neuroendocrine disease [37], and while the TRAMP mouse promoter is driven by androgens, the tumors derived from it are androgen-independent [15]. Despite these limitations, many of the findings were consistent with results reported in humans. As such, decreased AR and 5αR2 along with increased 5αR1 in tumors may promote PIN and prostate cancer development, thus warranting further investigation.

## Supporting information

S1 File This is data underlying the findings for genitourinary weight, raw and adjusted mean most severe and most common lesion scores, histopathological analysis (most severe and most common lesion), 5αR1, 5αR2, androgen receptor, Ki-67 and apoptosis, and 5αR1 in prostate vs. adjusted prostate most severe lesion score. (XLSX)

## References

[1] American Cancer Society. Cancer facts and figures 2017.

[2] Tien AH, Sadar MD. Androgen-responsive gene expression in prostate cancer progression In: Androgen-responsive genes in prostate cancer. Springer 2013;135–153.

[3] Grossmann ME, Huang H, Tindall DJ. Androgen receptor signaling in androgen-refractory prostate cancer. J Natl Cancer Inst. 2001;93(22):1687–1697. 11717329 10.1093/jnci/93.22.1687

[4] Andriole GL, Roehrborn C, Schulman C, Slawin KM, Somerville M, Rittmaster RS. Effect of dutasteride on the detection of prostate cancer in men with benign prostatic hyperplasia. Urology. 2004;64(3):537–543. doi: 10.1016/j.urology.2004.04.084 15351586 10.1016/j.urology.2004.04.084

[5] So AI, Hurtado-Coll A, Gleave ME. Androgens and prostate cancer. World J Urol. 2003;21(5):325–337. doi: 10.1007/s00345-003-0373-9 14586548 10.1007/s00345-003-0373-9

[6] Russell DW, Wilson JD. Steroid 5α-reductase: Two genes/two enzymes. Annu Rev Biochem. 1994;63(1):25–61. 7979239 10.1146/annurev.bi.63.070194.000325

[7] Luo J, Dunn TA, Ewing CM, Walsh PC, Isaacs WB. Decreased gene expression of steroid 5α- reductase 2 in human prostate cancer: implications for finasteride therapy of prostate carcinoma. Prostate. 2003;57(2):134–139. doi: 10.1002/pros.10284 12949937 10.1002/pros.10284

[8] Titus MA, Gregory CW, Ford OH, Schell MJ, Maygarden SJ, Mohler JL. Steroid 5α-reductase isozymes I and II in recurrent prostate cancer. Clin Cancer Res. 2005;11(12):4365–4371. doi: 10.1158/1078-0432.CCR-04-0738 15958619 10.1158/1078-0432.CCR-04-0738

[9] derströ TG, Bjelfman C, Brekkan E, Ask B, Egevad L, Norlén BJ, et al Messenger ribonucleic acid levels of steroid 5α-reductase 2 in human prostate predict the enzyme activity. J Clin Endocrinol Metab. 2001;86(2):855–858. doi: 10.1210/jcem.86.2.7224 11158057 10.1210/jcem.86.2.7224

[10] Bjelfman C, derströ TG, Brekkan E, Norlén BJ, Egevad L, Unge T, et al Differential gene expression of steroid 5α-reductase 2 in core needle biopsies from malignant and benign prostatic tissue. J Clin Endocrinol Metab. 1997;82(7):2210–2214. doi: 10.1210/jcem.82.7.4080 9215296 10.1210/jcem.82.7.4080

[11] Iehlé C, Radvanyi F, de Medina SGD, Ouafik LH, Gérard H, Chopin D, et al Differences in steroid 5α-reductase iso-enzymes expression between normal and pathological human prostate tissue. J Steroid Biochem Mol Biol.1999;68(5):189–195. 10416833 10.1016/s0960-0760(99)00030-8

[12] Nakamura Y, Suzuki T, Nakabayashi M, Endoh M, Sakamoto K, Mikami Y, et al In situ androgen producing enzymes in human prostate cancer. Endocr Relat Cancer. 2005;12(1):101–107. doi: 10.1677/erc.1.00914 15788642 10.1677/erc.1.00914

[13] Habib FK, Ross M, Bayne CW, Bollina P, Grigor K, Chapman K. The loss of 5α-reductase type I and type II mRNA expression in metastatic prostate cancer to bone and lymph node metastasis. Clin Cancer Res. 2003;9(5):1815–1819. 12738739

[14] Parekh DJ. Prostate cancer prevention with 5 alpha-reductase inhibitors. Recent Results Cancer Res. 2011;188:109–114. doi: 10.1007/978-3-642-10858-7_9 21253793 10.1007/978-3-642-10858-7_9

[15] Greenberg NM, DeMayo F, Finegold MJ, Medina D, Tilley WD, Aspinall JO, et al Prostate cancer in a transgenic mouse. Proc Natl Acad Sci U S A. 1995;92(8):3439–3443. 7724580 10.1073/pnas.92.8.3439 PMC42182

[16] Gingrich JR, Barrios RJ, Morton RA, Boyce BF, DeMayo FJ, Finegold MJ, et al Metastatic prostate cancer in a transgenic mouse. Cancer Res. 1996;56(18):4096–4102. 8797572

[17] Hurwitz AA, Foster BA, Allison JP, Greenberg NM, Kwon ED. The TRAMP mouse as a model for prostate cancer. Curr Protoc Immunol. 2001;Chapter 20:Unit 20.5 10.1002/0471142735.im2005s45 18432778

[18] Gingrich JR, Barrios RJ, Foster BA, Greenberg NM. Pathologic progression of autochthonous prostate cancer in the TRAMP model. Prostate Cancer Prostatic Dis. 1999;2(2):70–75. doi: 10.1038/sj.pcan.4500296 12496841 10.1038/sj.pcan.4500296

[19] Gingrich JR, Barrios RJ, Kattan MW, Nahm HS, Finegold MJ, Greenberg NM. Androgen-independent prostate cancer progression in the TRAMP model. Cancer Res. 1997;57(21):4687–4691. 9354422

[20] Kaplan-Lefko PJ, Chen TM, Ittmann MM, Barrios RJ, Ayala GE, Huss WJ, et al Pathobiology of autochthonous prostate cancer in a pre-clinical transgenic mouse model. Prostate. 2003;55(3):219–237. doi: 10.1002/pros.10215 12692788 10.1002/pros.10215

[21] Wang F. Modeling human prostate cancer in genetically engineered mice. Prog Mol Biol Transl Sci. 2011;100:1–49. doi: 10.1016/B978-0-12-384878-9.00001-7 21377623 10.1016/B978-0-12-384878-9.00001-7

[22] Berman-Booty LD, Sargeant AM, Rosol TJ, Rengel RC, Clinton SK, Chen CS, et al A review of the existing grading schemes and a proposal for a modified grading scheme for prostatic lesions in TRAMP mice. Toxicol Pathol. 2012;40(1):5–17. doi: 10.1177/0192623311425062 22021166 10.1177/0192623311425062 PMC4271830

[23] Opoku-Acheampong AB, Unis D, Henningson JN, Beck AP, Lindshield BL. Preventive and therapeutic efficacy of finasteride and dutasteride in TRAMP mice. PloS One. 2013;8(10):e77738 doi: 10.1371/journal.pone.0077738 24204943 10.1371/journal.pone.0077738 PMC3799703

[24] Conlon LE, Wallig MA, Erdman JW. Low-lycopene containing tomato powder diet does not protect against prostate cancer in TRAMP mice. Nutrition Research. 2015;35(10):882–890. doi: 10.1016/j.nutres.2015.07.003 26255194 10.1016/j.nutres.2015.07.003

[25] Shirakawa T, Okada H, Acharya B, Zhang Z, Hinata N, Wada Y, et al Messenger RNA levels and enzyme activities of 5 alpha-reductase types 1 and 2 in human benign prostatic hyperplasia (BPH) tissue. Prostate. 2004;58(1):33–40. doi: 10.1002/pros.10313 14673950 10.1002/pros.10313

[26] Thomas LN, Douglas RC, Lazier CB, Too CK, Rittmaster RS, Tindall DJ. Type 1 and type 2 5alpha-reductase expression in the development and progression of prostate cancer. Eur Urol. 2008;53(2):244–252. doi: 10.1016/j.eururo.2007.10.052 18006217 10.1016/j.eururo.2007.10.052

[27] Wako K, Kawasaki T, Yamana K, Suzuki K, Jiang S, Umezu H, et al Expression of androgen receptor through androgen-converting enzymes is associated with biological aggressiveness in prostate cancer. J Clin Pathol. 2008;61(4):448–454. doi: 10.1136/jcp.2007.050906 17720776 10.1136/jcp.2007.050906

[28] Thomas LN, Douglas RC, Lazier CB, Gupta R, Norman RW, Murphy PR, et al Levels of 5alpha reductase type 1 and type 2 are increased in localized high grade compated to low grade prostate cancer. J Urol. 2008;179(1):147–151. doi: 10.1016/j.juro.2007.08.155 17997435 10.1016/j.juro.2007.08.155

[29] Murti K, Butler LM, Sakko A, Ricciardelli C, Mitto TB, Octnick A, et al Androgen receptor levels during progression of prostate cancer in the transgenic adenocarcinoma of mouse prostate model. Medical Journal of Indonesia. 2010;19(1):5.

[30] Bassalyk LS, Petrova AS, Ermilova VD, Sokolova VK, Kushlinskiĭ NE. Relationship between androgen and estrogen receptors and clinico-morphological characteristics of prostatic cancer. Arkh Patol. 1983;45(2):23–28. 6847409

[31] Heinlein CA, Chang C. Androgen receptor in prostate cancer. Endocr Rev. 2004;25(2):276–308. doi: 10.1210/er.2002-0032 15082523 10.1210/er.2002-0032

[32] Harris TJ. Modulating self-reactive immune responses in auto-and tumor-immunity. ProQuest. 2009.

[33] Wikström P, Lindahl C, Bergh A. Characterization of the autochthonous transgenic adenocarcinoma of the mouse prostate (TRAMP) as a model to study effects of castration therapy. Prostate. 2005;62(2):148–164. doi: 10.1002/pros.20123 15389804 10.1002/pros.20123

[34] Xue L, Yang K, Newmark H, Lipkin M. Induced hyperproliferation in epithelial cells of mouse prostate by a Western-style diet. Carcinogenesis. 1997;18(5):995–999. 9163686 10.1093/carcin/18.5.995

[35] Nilsson S, Nordgren H, Eklöv S, Lögdahl M. Expression of Ki-67—a proliferation-associated antigen—in prostate cancer. Acta Oncol. 1991;30(2):177–179. 2029402 10.3109/02841869109092346

[36] Zeng L, Kyprianou N. Apoptotic regulators in prostatic intraepithelial neoplasia (PIN): value in prostate cancer detection and prevention. Prostate Cancer Prostatic Dis. 2005(1);8:7–13. doi: 10.1038/sj.pcan.4500757 15477876 10.1038/sj.pcan.4500757

[37] Chiaverotti T, Couto SS, Donjacour A, Mao JH, Nagase H, Cardiff RD, et al Dissociation of epithelial and neuroendocrine carcinoma lineages in the transgenic adenocarcinoma of mouse prostate model of prostate cancer. Am J Pathol. 2008;172(1):236–246. doi: 10.2353/ajpath.2008.070602 18156212 10.2353/ajpath.2008.070602 PMC2189611

